# Streptokinase for Treatment of Thrombotic Disorders: The End? Or the End of the Beginning?

**Published:** 2018-05

**Authors:** Farzin Roohvand

**Affiliations:** Virology Department, Pasteur Institute of Iran, Tehran, Iran

**Fig. 1:**
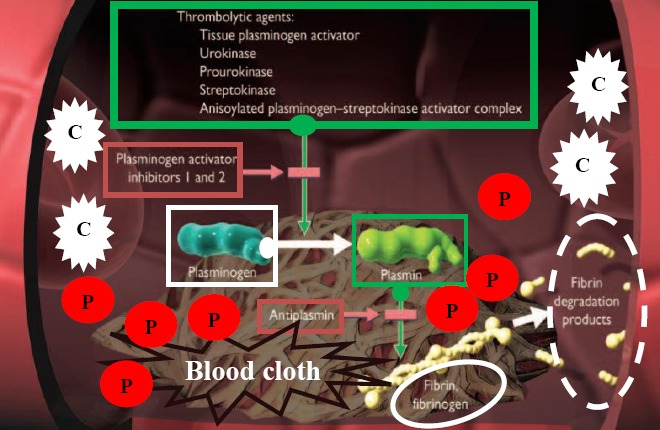
Schematic diagram of blood clot (thrombus) lysis by thrombolytic agents via plasminogen activation in a typical vein. White blood cells are indicated by “C”, and platelets entrapped within blood cloths are shown by “P”.

Thrombotic disorders, such as myocardial infarction, ischemic stroke, peripheral arterial disease, deep venous thrombosis, pulmonary embolism, or other embolic diseases that are responsible for worldwide mortality and morbidity, are manifestations of the formed thrombi by blood clots during a pathologic blood coagulation process. Once thrombi are formed, the only way to resolve the blood clot is treatment with specific thrombolytic agents, so called plasminogen activators (PAs), such as tissue-PAs (tPAs), urokinase, and streptokinase (SK). PAs convert the endogenous human plasminogen (HPG: the inactive proenzyme) to plasmin (HPM: the active enzyme), which cleaves the fibrin network of the blood clot, a process known as fibrinolysis (Fig. 1).

Among PAs, SK is the first drug approved for clinical applications and has been used in the treatment of thrombotic disorders since 1950s. However, there were major advances in its application in 1960s while the drug was produced and provided by Kabi Pharmacia (Sweden). Currently, SK is being manufactured by a number of pharmaceutical companies throughout the world under the trade names of: kabikinase, streptase, indikinase, and varidase. Although SK has saved thousands of lives from its first clinical utilization to date, its use is accompanied with some adverse side effects. In fact, SK is a bacterial protein produced by Lancefield groups A, C, and G *β-hemolytic streptococci*. Historically, the commercial source of the drug was a group C isolate (*Streptococcus equisimilis* H46A). As a microbial protein, SK is highly immunogenic and induces the elicitation of antibodies in humans that can inactivate the drug. Therefore, SK cannot be re-administered for at least six months after its first application and is usually considered for the first-line therapies in thrombotic disorders. Accordingly and as a result of prior streptococcal infections, administration of SK might also result in the risk of allergic reaction in humans with severe, even fatal, anaphylactic responses. In addition, SK is considered as a non-fibrin specific PA, i.e. not targeted to the blood clot. Therefore, its systematic application might induce hemorrhage. Despite all these shortcomings, SK was globally recognized as a life-saving and a must-be available drug in emergency and intensive care units of medical centers. However, by the emergence of recombinant DNA technology and the availability of recombinant tPAs produced from human DNA in 1980s, the human compatible, non-immunogenic and fibrin-specific tPA with less adverse effects was supposed to completely take the place of SK for treatment of thrombotic disorders. Indeed, SK was gradually removed from pharmacopeia of several developed countries and replaced with tPA, though almost all clinicians throughout the world believed that there will be no more place for SK in fibrinolytic therapy.

Interestingly later, the results of different global double-blinded trials for these two PAs did not prove any clear preferences for either drug. Moreover, last generations of genetically modified tPA, such as tenecteplase (TNK), retained higher stability, half-life, and fibrin-specific properties but could not completely justify their high price compared to SK therapy (which is around ten times) for almost the same mortality rates and bleeding complications. Thus, SK proved to be at least the most cost-effective fibrinolytic drug. As a result, considering the unavailability of a low-priced generic or biosimilar tPA, SK continued to be the drug of choice for thrombolytic therapy in developing countries, and accordingly, continuous efforts were undertaken to improve its fibrinolytic characteristics as a thrombolytic drug (i.e. access to a fibrin-specific, less immunogenic SK with higher *in vivo* half-life). To this end and following the identification of structure and function of SK (i.e. the role of substrate interacting sites [exosites] and critical amino acids [hot spots] within the SK [α, β, and ϒ] domains) on its PAs properties, several approaches were considered, which includes: i) Genetic manipulations such as amino acid substitution/addition/deletion. For instance, truncated SKs deprived from residues 1-59 (rSKϪ1-59) showed enhanced fibrin-specific HPG activities and lowered immunogenicity compared to full-length SK, while substitution of Lys59 and/or Lys386 for glutamine increased the half-life. ii) Chemical modifications such as homogenous/site-specific PEGylation, glycosylation, or acylation (like anisoylated plasminogen-SK activator complex; APSAC) have shown increased half-life and stability accompanied with reduced immunogenicity. iii) Domain fusions through production of chimeric and conjugated SK proteins like conjugation of SK with fibrin or platelet-specific monoclonal antibodies (to increase their clot specificity) or hirudin (the most potent natural thrombin inhibitor) to inhibit thrombin activity or conjugation with dendrimers. iv) Liposomal entrapment of SK or encapsulation in PEG or chitosan nanoparticles to enhance half-life and stability and to reduce the immunogenicity as well as better clot penetration properties. In addition, SK is a heterogeneous protein, and SKs produced by even different strains in the same Lancefield group show various levels of amino acids variability accompanied by altered PA properties. Differences in PA properties of *streptococcal* culture supernatants (SCS) have been traditionally used to identify superior producer strains and SK genes (*skc*) for recombinant SK production. However, more exact studies have indicated no activity correlation for streptococcal culture supernatant and recombinant SKs. Accordingly, PCR-restriction fragment length polymorphism (PCR/RFLP)-based analysis of β-domain variable region of *skc* could not precisely categorize *skc* alleles for their PA properties. However, recently, phylogenetic tree analyses of the same region of SK and identification of SK clusters could better correlate these searched properties (i.e. SKs with better fibrin-specific fibrinolytic properties), thus brightening the path for the way that desired SK genes for efficient thrombolytic therapy should be searched. In this context, more recently, screening programs for isolation of SK-producing *β-hemolytic streptococci* resulted in identification of two group G streptococci with high fibrin-dependent plasminogen activities that showed novel altered amino acids as potential hot spots. Identification of hot spots in SK structure and recent advances and potential application of computer and matrix-based molecular modeling pave the way for designing and introducingsuperior SKs.

Although most of the studies belong to *in vitro* approaches, the increased rate of the research and reports for improvement of SK in recent years indicates the amount of interest on improving and reviving this first and most exploited thrombolytic drug. Therefore, it is not the end but the end of the beginning for further research and studies on SK to gain the best cost-effective drug for treatment of thrombotic disorders. The show must go on.

**More details in:**

***Towards a superior streptokinase for fibrinolytic therapy of vascular thrombosis***. M Keramati et al. 2013. Cardiovasc Hematol Agents Med Chem; Vol. 11, pp. 218-229.

***Thrombolytic and fibrinogenolytic properties of bioconjugate streptokinase-polyamidoamine dendrimers in vitro***. LI Mukhametova et al. 2017. Thromb Res; Vol. 154, pp. 50-52.

***Sequence and kinetic analyses of streptokinase from two group G streptococci with high fibrin-dependent plasminogen activities and the identification of novel altered amino acids as potential hot spots***. M Keramati et al. 2017. Biotechnol Lett; Vol. 39, pp. 889-895.

***Pitfalls in screening streptococci for retrieving superior streptokinase (SK) genes: no activity correlation for streptococcal culture supernatant and recombinant SK***. M Keramati et al. 2013. J Ind Microbiol Biotechnol; Vol. 40, pp. 151-158.

***Liposomes for the delivery of streptokinase***. L Kumar et al. 2017. Ther Deliv; Vol. 8, pp. 855-866.

***PCR/RFLP-based allelic variants of streptokinase and their plasminogen activation potencies***. M Keramati et al. 2012. FEMS Microbiol Lett; Vol. 335, pp. 79-85.

**Translational initiatives in thrombolytic therapy**. ME Klegerman et al. 2017. Front Med; Vol. 11, pp. 1-19.

